# Thermochemical Transition in Low Molecular Weight Substances: The Example of the Silybin Flavonoid

**DOI:** 10.3390/molecules27196345

**Published:** 2022-09-26

**Authors:** Costas Tsioptsias, Christina Spartali, Sotirios I. Marras, Xanthi Ntampou, Ioannis Tsivintzelis, Costas Panayiotou

**Affiliations:** 1 Department of Chemical Engineering, Aristotle University of Thessaloniki, University Campus, 54124 Thessaloniki, Greece; 2 Department of Biochemistry and Biotechnology, University of Thessaly, 41500 Larissa, Greece

**Keywords:** silybin, thermochemical transition, melting, decomposition, simultaneous, hydrogen bonding, thermal stability

## Abstract

Silybin is a complex organic molecule with high bioactivity, extracted from the plant Silybum. As a pharmaceutical substance, silybin’s bioactivity has drawn considerable attention, while its other properties, e.g., thermodynamic properties and thermal stability, have been less studied. Silybin has been reported to exhibit a melting point, and values for its heat of fusion have been provided. In this work, differential scanning calorimetry, thermogravimetry including derivative thermogravimetry, infrared spectroscopy, and microscopy were used to provide evidence that silybin exhibits a thermochemical transition, i.e., softening occurring simultaneously with decomposition. Data from the available literature in combination with critical discussion of the results in a general framework suggest that thermochemical transition is a broad effect exhibited by various forms of matter (small molecules, macromolecules, natural, synthetic, organic, inorganic). The increased formation of hydrogen bonding contributes to this behavior through a dual influence: (a) inhibition of melting and (b) facilitation of decomposition due to weakening of chemical bonds.

## 1. Introduction

Milk thistle (*Silybum marianum*) seeds have been used for thousands of years as herbal remedies for the treatment of various ailments. The extract from this medicinal plant, known as silymarin, contains several flavonolignans (silybin, isosilibinin, silychristin, isosilychristin, and silydianin) and displays remarkable antioxidant, anti-inflammatory, immunomodulatory, and hepatoprotective properties [1]. The main component of the silymarin complex is silybin, which comprises a quasiequimolar mixture of two diastereomers: silybin A and silybin B. Silybin has a molecular formula of C_25_H_22_O_10_ and its structure is characterized by the amalgamation of a flavonoid unit (taxifolin) and a phenylpropane unit (coniferyl alcohol) [2]. It shows low water solubility, less than 50 µg/mL, due to its highly hydrophobic and nonionizable structure, which highly affects its bioavailability [3]. However, silybin exhibits important biological effects which have been confirmed in various studies [3,4].

Other biological properties of silybin, e.g., its physicochemical and thermodynamic characterization, have been underexplored. In some cases, thermodynamic properties of silybin in solutions have been reported, e.g., dissociation constant in aqueous solutions [5], or solubility in poly(ethylene glycol) (PEG) [6], poly(vinylpyrrolidone) (PVP) [7], and dextran solutions [8]. However, for solvent-free processes, the knowledge and understanding of the solid-state properties of the materials is of primary importance. Furthermore, properties of antioxidant substances, such as their thermal stability, are very important for the evaluation of antioxidant potential, especially for high-temperature applications related to thermo-oxidation, e.g., pasteurizing, sterilizing, extrusion processing, etc. Various natural antioxidants including gallic acid, α-tocopherol, caffeic acid, and ferulic acid have been evaluated for their thermal stability and their potential as antioxidants for biodiesel protection [9]. For high-temperature applications, the knowledge of phase transition points, e.g., melting point, is important because the formation of liquid phase can measurably affect reaction rates [10]. Moreover, the biological antioxidant activity of a substance may be limited by reduced availability or poor solubility. For poorly soluble hydrophobic drugs, solid dispersion is a promising approach for improving bioavailability and/or controlling dissolution rate [11], and various substances, e.g., PEG, can be used as drug carriers. For such cases and for designing new drug formulations, the knowledge of thermodynamic properties is important. In this context, the heat capacity of solid (dispersion) mixtures of silybin with PVP [11] and sodium cholate [12] have been measured, and the melting point and heat of fusion of solid dispersions of silybin with PEG 6000 [13] and with poloxamer 188 [14] have been reported. In all these cases [11,12,13,14], as well as in various other cases [6,7,8], [3,15,16] silybin has been reported to exhibit a melting point; depending on the diastereomers A or B, the melting point is 162–163 °C or 158–160 °C, respectively [15]. In different cases, the heat of fusion of silybin has been reported to be 161 J/g [14] or 93 J/g [6,7,8,13]. To the best of our knowledge, only in two cases were mass losses realized in a temperature range very close to the melting temperature range of silybin, and these were attributed to water evaporation [11,12]. In an interesting review article about the chemistry of silybin, it was reported that breakage of its skeleton occurs either due to strong base or by prolonged heating above 100 °C. However, the same melting temperatures (158–163 °C) as in the other references were also reported [17]. The two pure diastereomers, namely, silybin A and silybin B, were reported to have close melting points equal to 158–160 °C and 157–159 °C, respectively [17].

Silybin is obtained from silymarin by extraction with methanol, and it commonly contains isomers as impurities [17,18]. General flavonoids, e.g., quercetin and rutin [19], and other similar molecules (phenolic molecules with increased numbers of OH groups), e.g., gallic acid (3,4,5-trihydroxybenzoic acid) [20], exhibit extensive polymorphism. In a study regarding the thermal behavior of gallic acid, although a melting point was reported, the value for the heat of fusion was not reported due to realization or suspicion of decomposition [21]. For quercetin dihydrate, it was reported that decomposition related to water removal occurs prior to melting [19].

Furthermore, it has been recognized that modelling for such molecules and other similar pharmaceuticals has shown poor efficiency, and prediction of their thermodynamic properties, including melting point, is difficult [22,23]. One common characteristic of such molecules is the increased capacity for hydrogen bonding. The effect of hydrogen bonding on the thermal transitions of matter, i.e., inhibition of melting and boiling, is well known. However, hydrogen bonding also has the effect of weakening chemical bonds [24].

In addition to reported poor efficiency in predicting the melting point of pharmaceuticals, the appearance of some “melting” during decomposition implies peculiarities in the thermal behavior of pharmaceuticals. In general, preparation of drugs through high temperature melt processing (to avoid the use of solvents) is usually constrained by drug decomposition [13]. For some solids, it is known that decomposition is accompanied by melting [25]. A special branch of decomposition kinetics dedicated to this effect has been developed, and is known as Bawn kinetics [25] after the scientist who developed it. The Bawn model focuses on the decomposition time profile and considers simultaneous melting, mainly in terms of solubility (due to formation of liquid phase) between reactants and products [25]. Also, it has been proposed that the formation of liquid state during a reaction of initially solid substances may result in increased reaction rates [10]. The Bawn model has been used for organic explosives [25] that exhibit such an effect, e.g., octahydro-1,3,5,7-tetranitro-1,3,5,7-tetrazocine [26], and have been reported to exhibit simultaneous decomposition and melting. Interestingly, the concept of Bawn kinetics has also been applied to pharmaceuticals [25].

Similar effects of simultaneous phase transition and decomposition have been reported for lithium potassium tartrate [27] and potassium perchlorate [28], and the effect is suspected to be responsible for the deviations in the reported values of the melting point of succinic acid [29]. Furthermore, similar peculiarities in thermal behavior, i.e., decomposition prior to melting, have been reported for certain hydrated flavonoids such as quercetin dihydrate and rutin dihydrate [19]. Independently from the above, an analogous effect was recently found to occur in some polymers. More specifically, during softening in these polymers which had erroneously been attributed to melting, some minor decomposition was found to occur, e.g., for cellulose esters and poly(vinyl alcohol) (PVA) [30,31]. As this effect is different from neat melting, it deserves a distinct name and was initially termed “glass chemical transition” [30]; after recognizing its more general nature beyond glass transition, it was termed “thermochemical transition” [31].

The scope of this current work is to provide evidence for the broad character of thermochemical transition. The thermal behavior of silybin was examined as a case study. Insights for the explanation of this behavior within a general framework are provided.

## 2. Results

**Figure 1:**
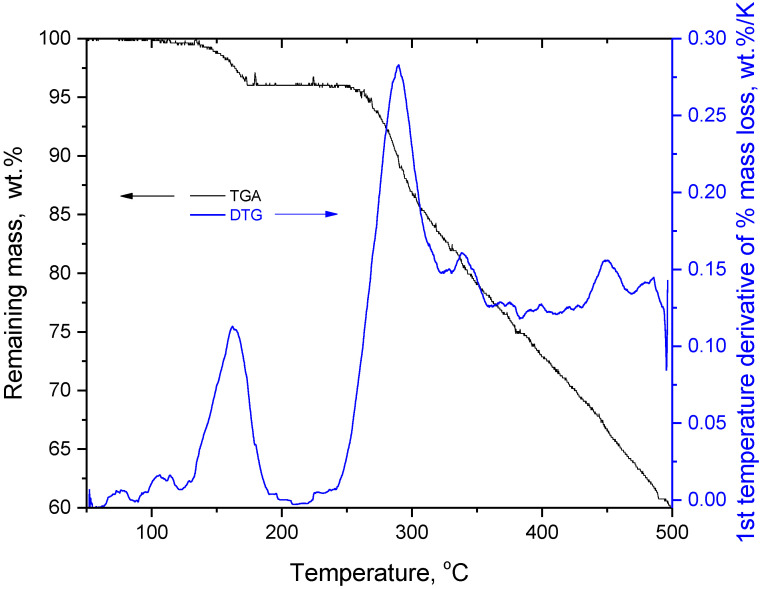
TGA and DTG (first temperature derivative of % mass loss) curves of silybin in the temperature range 50–500 °C.

The TGA and first temperature derivative of percentage mass loss (DTG) curves of silybin are presented in Figure 1. The TGA curve is in agreement with the TGA curves of silybin reported in the literature [11].

**Figure 2:**
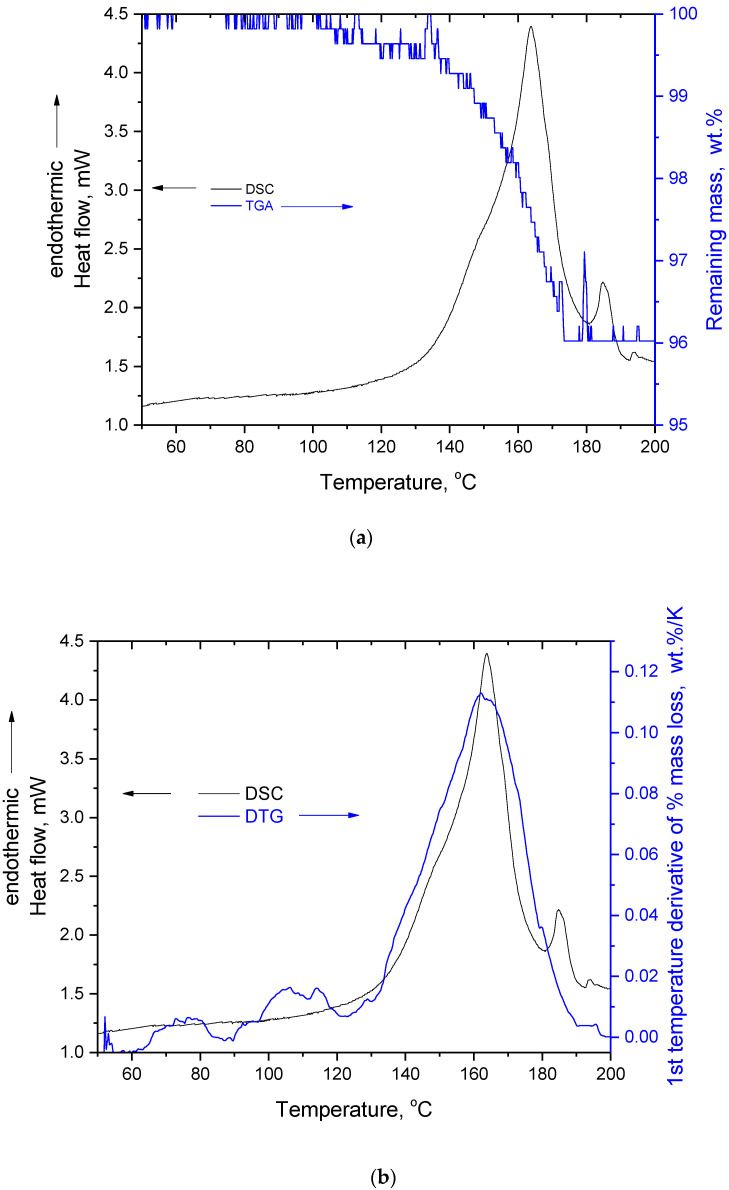
(**a**) DSC and TGA curves of silybin and (**b**) DSC and DTG (first temperature derivative of % mass loss) curves of silybin.

As can be seen in Figure 1, there was a minor mass loss at temperatures below 175 °C and after that there was no (detectable) mass loss up to 250 °C, where the main decomposition of silybin occurred. In the literature, the mass loss of the first stage has been attributed to water evaporation [11]. However, this mass loss occurred in the same temperature range as the endothermic peak detected in the DSC trace of silybin (Figure 2a). The maximum of the DSC peak was located at 163.7 °C, in very good agreement with the reported values of silybin’s melting point (see introduction for references).

At the end of the major peak (around 180 °C), a minor endothermic peak was detected. This peak was also detected in a second independent DSC repetition measurement (not shown) and might be attributed to impurities. In Figure 2b, the DSC trace is presented along with the curve of the first temperature derivative of % mass loss (DTG). A remarkable coincidence and identification between these two curves can be recognized. Specifically, the temperature range where the mass loss rate reached its maximum (see the TGA curve in Figure 2b) perfectly coincided with the temperature range where the heat absorption rate reached its maximum (see the DSC curve in Figure 2b). This identification clearly indicates that the mass loss detected in TGA and the endothermic phenomenon detected in DSC were highly correlated. Even if this mass loss is attributable to water evaporation, the value for the heat of fusion is erroneous because the heat of vaporization of water was included in the heat measured by DSC. However, the mass loss cannot be attributed exclusively to water evaporation; the reasons for this are discussed below.

In Table 1 the heat measured by DSC is expressed as specific heat, according to two approaches. The first approach is to divide the heat measured by DSC by the overall mass of the sample used in the DSC measurement (2.7 mg), or in other words to express it as “heat of fusion”. Since it has been shown that the heat and the mass loss are highly connected, the second approach is to treat it as heat of desorption, i.e., to divide the heat measured by DSC not by the overall mass of the sample, but by the mass loss (~0.1 mg, this value corresponds to 4 wt.% decomposition of 2.7 mg of the initial sample mass). This specific heat is termed as the specific heat of thermochemical transition. These values, along with the temperature range used for the integration of the DSC peak, are presented in Table 1.

The value of the calculated “heat of fusion” is comparable and of the same order of magnitude as reported values for silybin’s heat of fusion, e.g., 93 and 161 J/g (see introduction for respective references). However, as was reported for polymers [30,31] and gallic acid [21], the physical meaningfulness of this “heat of fusion” is questionable due to mass loss and decomposition. The value of the specific heat of thermochemical transition is higher than the heat of water desorption. More precisely, the heat of water desorption depends on the water content, and reaches its maximum value for very low water content (desorption of the monolayer), which typically approaches the value of water’s heat of vaporization (~2200 J/g). For example, the maximum values of heat of water desorption from lignite, montmorillonite, and apple have been reported to be equal to 453.9 J/g [32], 2222.2 J/g [33], and 1983.3 J/g [34], respectively. It should be stressed that, as reported recently [35], the value for the specific heat of thermochemical transition is characterized by high uncertainty despite the rather low uncertainties resulting from the instruments’ sensitivity (± 0.01 mW for DSC and ± 0.01 mg for TGA). Briefly, the high uncertainty arises mainly from two factors. The first factor is that some decomposition products may not vaporize in the temperature range of the thermochemical transition (due to high molecular weight or increased hydrogen bonding, or a combination of these two). Thus, DSC detects the heat required for the formation of all the decomposition products; however, not all of these contribute to the mass loss detected by TGA. Therefore, when calculating specific heat there may not be a full correspondence between heat and mass loss. The second factor is that the heat capacity baseline (in practice, the baseline used for the integration of the DSC peak) may change in a non-typical manner. More precisely, the heat capacity (mass times specific heat capacity) of a sample typically increases with temperature. When integrating a melting peak, the mass of a sample is normally constant and a linear increase of specific heat capacity can be assumed. Thus, the baseline used for the integration is a line with a slope towards the endothermic direction in the DSG graph. In the case of silybin, the sample mass was reduced (causing the baseline to shift towards the exothermic direction) and the specific heat capacity changed due not only to temperature, but also due to the chemical reactions. Thus, it was not clear which baseline should be used for the integration of the DSC peak, which introduced additional uncertainty in the estimation of heat measured by DSC. The determination of the proper baseline in such a case is a difficult task and warrants dedicated study. In any case it should be stressed that the value of the specific heat of thermochemical transition is characterized by high uncertainty and should be treated accordingly, i.e., it should not be considered an accurate value, but only as an order of magnitude for comparison with the specific heat of fusion.

Thus, the high value (order of magnitude) of the specific heat of thermochemical transition suggests chemical bond breaking. The dissociation energy of chemical bonds is in the range 200–1000 kJ/mol, e.g., the isolated C-C bond has dissociation energy of 610 kJ/mol [36], while an average value of C-C dissociation energy in various organic molecules was found to be 347 kJ/mol [37]. Other common chemical bonds have average values of dissociation energies in the range 200 to 1000 kJ/mol [37]. Thus, an average value of 600 kJ/mol can be considered representative of the dissociation energy for the majority of chemical bonds. In order to express the specific heat of thermochemical transition in kJ/mol, the value in J/g must be multiplied by some molecular weight; the question is which molecular weight should be used. If the molecular weight of silybin (482 g/mol) is used, then a value of 1375 kJ/mol is obtained. However, the quantity (g) of decomposed mass relates to the decomposition products, which have much lower molecular weight. By assuming that the only decomposition product is water and using its molecular weight, then the obtained value for the specific heat of thermochemical transition is 51 kJ/mol. Of course, the decomposition products may have such a low molecular weight, but decomposition products with higher molecular weight are also possible. Thus, if the average value of these two extreme cases (1375 and 51 kJ/mol) is calculated, then, the obtained value is 713 kJ/mol, remarkably close to the abovementioned values for chemical bond dissociation energies. It must be stated that these calculations lack accuracy and are characterized by large uncertainties. However, they were carried out to indicate that the experimental value of the specific heat of the thermochemical transition is of the same order of magnitude as chemical bond dissociation energies, suggesting the phenomenon of decomposition. Further evidence for decomposition of silybin during its “melting” is provided by the FTIR analysis, presented below.

**Figure 3:**
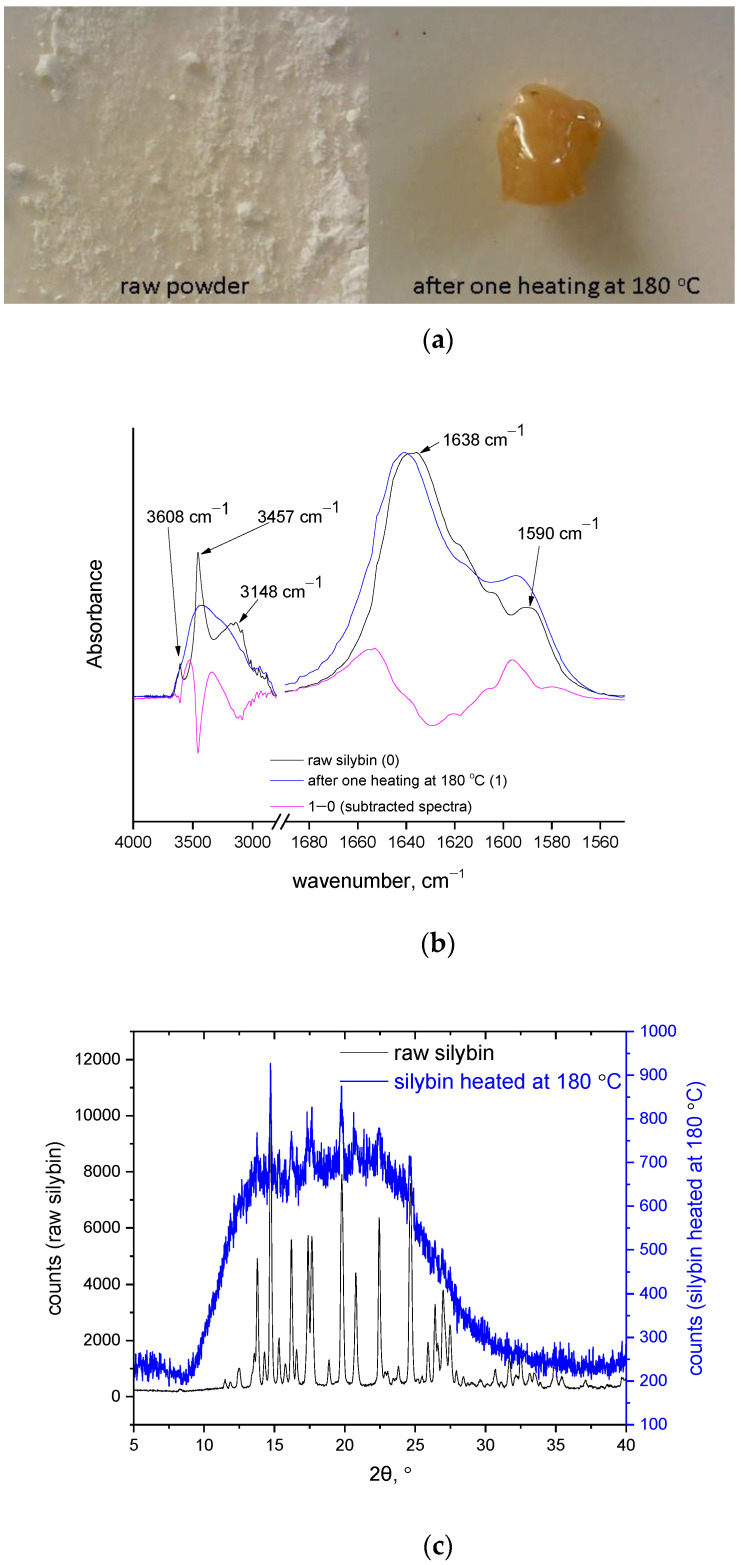
(**a**) Digital microscope images of silybin before and after heating to 180 °C, (**b**) FTIR spectra of raw and heated silybin samples and their subtracted spectra, and (**c**) XRD patterns of silybin before and after being heated to 180 °C.

In the TGA curve of Figure 2a, it can be seen that at 180 °C the mass loss had ended, and in the DSC trace it can be seen that at the same temperature the (major) endothermic peak was almost complete. Thus, if the endothermic peak in DSC had arisen from melting of silybin, and if the mass loss up to 180 °C was caused by water evaporation, then no signs of alteration to its chemical structure should be detected when heating silybin to 180 °C. For this reason, a TGA measurement of a raw silybin sample was performed in the temperature range 50–180 °C (curve not shown). The same mass loss (4 wt.%) was detected as in the TGA presented in Figure 2a. Then, this heated sample was removed from the TGA pan and was examined macroscopically and by FTIR. Note that the silybin grains of the raw sample formed a single piece after heating, and extensive coalescence of the grains occurred, as can be seen in Figure 3a, indicating that softening indeed occurred during heating up to 180 °C. However, from the same figure it is apparent that simply heating silybin to 180 °C was more than sufficient to cause considerable alteration of its color (from light yellow in the raw sample, the color became dark yellow-brown in the heated sample). Such color alteration cannot be attributed to water evaporation, suggesting on the contrary that alterations in the chemical structure were in turn responsible for alterations in light absorption by the sample. Changes in the chemical structure of silybin after heating to 180 °C were clearly verified by the FTIR spectra of the raw and the heated samples, presented in Figure 3b.

The peaks at around 3457 cm^−1^ and 3608 cm^−1^ are attributable to free or loosely bound hydroxyl vibrations, and the peak around 3148 cm^−1^ to bounded hydroxyl groups [14], although overlap with aromatic C-H vibrations is possible [38]. In any case, considerable alterations in this region (3000–4000 cm^−1^) were observed after one heating at 180 °C. In the subtracted spectra (spectrum after heating minus spectrum before heating) clear negative peaks were detected, suggesting decreases of hydroxyl content and aromatic C-H content. A negative peak was observed at 1638 cm^−1^ assigned to (conjugated) C=O vibration [14]. Finally, positive peak at around 1590 cm^−1^ assigned to C=C stretching [38] was detected in the subtracted spectra. Note that these differences were also observed in the non-subtracted spectra, e.g., the increased intensity of the 1590 cm^−1^ peak in the heated sample was obvious compared to that of the raw sample. These results suggest a perceptible alteration of the chemical structure of silybin after heating, and cannot be attributed simply to evaporation of impurities.

Considerable changes in the crystalline structure, as observed in the XRD patterns (Figure 3c), also point in the same direction. As can be seen in Figure 3c, the raw silybin powder exhibited a pattern typical of highly crystalline materials, with many sharp peaks. The baseline was fairly straight and there was no sign of amorphous regions. The XRD pattern of the raw silybin was generally similar to other reported XRD patterns for silybin from different providers and with slightly different purities [39,40]. Thus, the silybin used in this study was a typical silybin sample. After heating to 180 °C, the XRD pattern showed a pattern typical of highly amorphous material, with only one very broad peak. Some sharp peaks were detected within this broad peak, at the same angles as for raw silybin. Thus, it can be concluded that decomposition up to 180 °C causes severe disruption in the crystal lattice of silybin and leaves very few crystals. Based on these findings, it is evident that the endothermic peak in the DSC trace of silybin did not arise from neat melting but from thermochemical transition (simultaneous softening and decomposition). One interesting question that arises concerns the reason for the absence of (detectable) mass loss between the end of thermochemical transition (180 °C) and initiation of decomposition (250 °C). The increase of C=C content, as indicated by FTIR, seems to be in agreement with the darker color of the heated sample, e.g., the brown color of oxidized poly(propylene) arose from the formation C=C bonds [41,42]. Also, it should be taken into account that the structure of silybin is similar to char structures (of polycyclic and heterocyclic compounds), thus the formation of increased char during thermal decomposition of silybin may be expected. Increased char formation was detected at the end of the TGA measurement at 500 °C. As can be seen in Figure 1, at 500 °C silybin retained 60 %wt. of its initial mass. From the DTG curve shown in Figure 1, it can be seen that the maximum mass loss rate occurred at 280 °C, which was 30 °C above the decomposition initiation temperature (250 °C). These findings suggest increased char formation. More specifically, due to its structure, silybin can more easily result in char formation compared with linear polymers. Thus, soon after initiation of decomposition (250 °C) the rapid formation of thermally stable char regions caused a rapid decrease in decomposition rate at 280 °C. A similar effect with a smaller extent was apparent during decomposition in the thermochemical transition. The minor decomposition during thermochemical transition resulted in char-like structures that were more thermally stable than raw silybin. These new structures were responsible for the dark color and the positive C=C peaks revealed by FTIR in the sample heated to 180 °C, and for the absence of mass loss in the temperature range 180 to 250 °C. The following discussion presents an additional explanation for the absence of mass loss in the range 180 to 250 °C, among other issues.

As discussed in the introduction section, the vast majority of substances exhibiting thermochemical transition share a particular common characteristic. Such substances (see introduction section for references) include cellulose esters, PVA, succinic acid, lithium potassium tartrate, quercetin dihydrate, gallic acid and, as presented in this study, silybin. The common characteristic of all these substances is their high capacity for formation of hydrogen bonds. The effect of hydrogen bonding on the behavior of cellulose esters and PVA has been discussed [30,31], in comparison with cellulose and chitin that are known not to exhibit detectable thermal transitions prior to decomposition. Briefly, the hydrogen bonding network is responsible for rigidity in the structure and inhibits melting. It seems that in small complex molecules, including polymers, increased hydrogen bond formation is an important factor determining their inability to melt. Here, we discuss how hydrogen bonding, in addition to inhibiting melting by keeping molecules close to each other, also has the effect of weakening chemical bonds and thus enabling decomposition. This double effect of hydrogen bonding plays an important role in thermochemical transition. The vibrational wavenumber, $\tilde{\nu }$, of the molecular oscillator is given by the following equation [43]:

(1) $$\tilde{\nu }=\frac{1}{2\pi c}\sqrt{\frac{k}{\mu }}$$

where *c*: speed of light; *k*: the force constant of the chemical bond between two atoms of mass *m*_1_ and *m*_2_; *μ*: the reduced mass, i.e.:

(2) $$\mu =\sqrt{\frac{m_{1}\times m_{2}}{m_{1}+m_{2}}}$$

The force constant is an accepted way to express the strength of a chemical bond [44,45]. In general, it is known that hydrogen bonding reduces the O-H stretching vibration wavenumber [38]. More specifically, hydrogen bonding alters the length (and/or the angle) of the chemical bond, and consequentially the force constants of free and bounded OH groups are different. The stretching of the chemical bond due to hydrogen bonding leads to its weakening, which in turn is translated to lower force constant that ultimately results in the lower vibrational wavenumber of bounded OH groups. Of course, the force constant (and thus the vibrational wavenumber) also depends on other factors, including inter- and intra-molecular hydrogen bonding and neighboring chemical bonds. For example, the force constant of an OH group that forms a hydrogen bond with an oxygen atom of another OH will be different from one with an oxygen atom belonging to a C=O or C-O group, etc. Thus, for various reasons, with hydrogen bonding important among these, the same chemical bond may exhibit variations of strength. To quantify this variation, Equation (1) may be used. Specifically, the OH band of silybin covers a range of wavenumbers, i.e., from 3100 cm^−1^ for highly shifted (strongly bounded) to 3600 cm^−1^ for free OH groups. By substituting these values in Equation (1) and dividing by terms it follows that:

(3) $$\frac{3600}{3100}=\frac{\frac{1}{2\pi c}\sqrt{\frac{k_{free}}{\mu }}}{\frac{1}{2\pi c}\sqrt{\frac{k_{shifted}}{\mu }}}\Rightarrow \;\frac{k_{shifted}}{k_{free}}=0.74$$

Thus, the strength of a highly shifted OH group is about 0.74 of the strength of a free OH group. These highly shifted OH groups (weakened up to 26%) are likely to be involved in decomposition during thermochemical transition.

**Figure 4:**
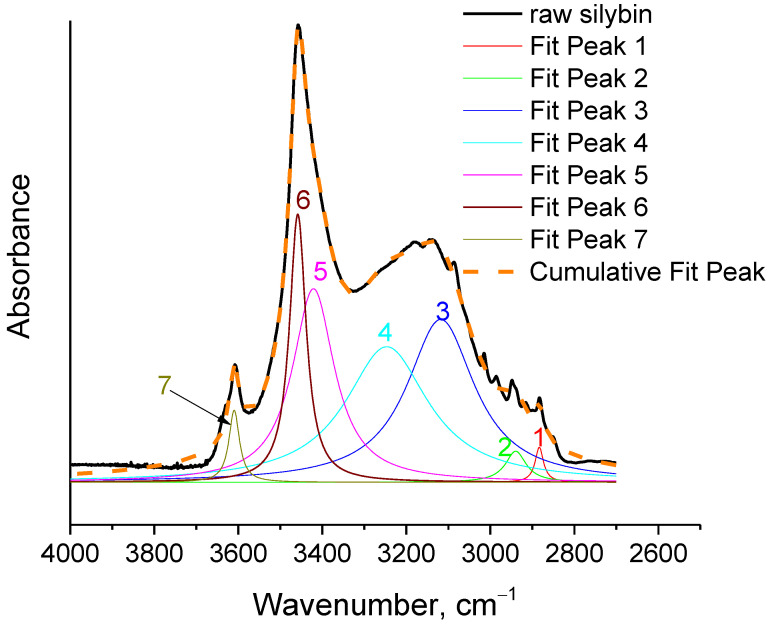
Curve fitting (with Lorentzian peaks) of the spectrum of raw silybin in the region 2700–4000 cm^−1^.

In order to further deepen the understanding obtained in the above discussion, curve fitting (with Lorentzian peaks) was performed in the spectrum of raw silybin in the region 2700–4000 cm^−1^, and is illustrated in Figure 4. The wavenumber and the percentage areas of the fitted peaks are presented in Table 2. It should be noted that the fitted peaks one, two, and three were not taken into account in the normalization of the areas, since these peaks were assigned to vibrations other than O-H stretching. As can be seen in Figure 4 and Table 2, the fitting procedure revealed the two expected contributions in the peak at 3147 cm^−1^. As discussed in the previous section, highly shifted OH groups contributed to this peak, overlapping with the contribution from aromatic C-H stretching. The fitted peak three (at 3118 cm^−1^) represents the C-H contribution, while fitted peak four (at 3247 cm^−1^) represents the contribution of highly shifted OH. The latter represented 46% of the OH group vibrations. For the peak at 3608 cm^−1^, one fitted peak was sufficient to achieve proper fitting (fitted peak seven). This peak represented only the 3% of the overall OH vibrations.

**Figure 5:**
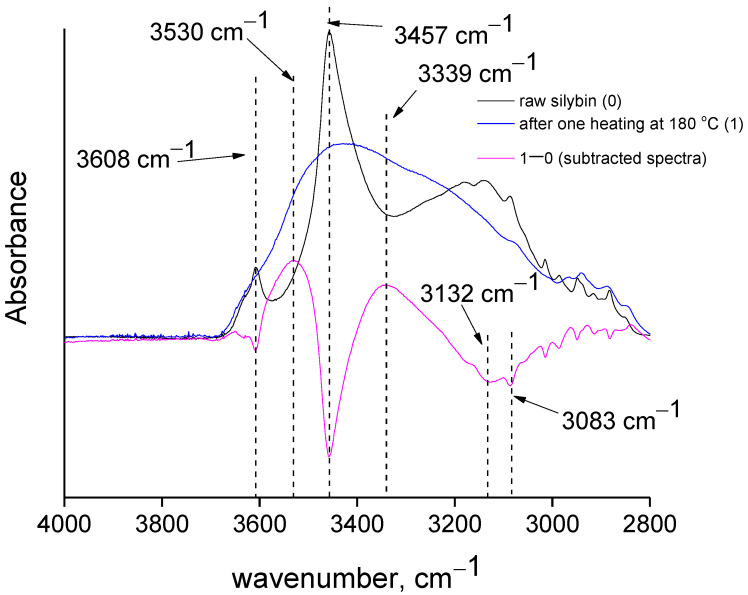
FTIR spectra of raw and heated silybin samples and their subtracted spectrum in the region 2800–4000 cm^−1^.

For the main peak at 3457 cm^−1^, two peaks were again required to ensure proper fitting (peaks five and six). The cumulative percentage area of these peaks was 33 + 18 = 51% ≈50%. From the results presented in Table 2, it can be concluded that a large portion of OH groups in raw silybin are strongly bounded, and thus many of the OH groups are involved in weak chemical bonds. In Figure 5, the spectra shown in Figure 3 are presented on a different scale, focusing on the O-H stretching region. In agreement with the discussion above, negative peaks at around 3132 cm^−1^ and at 3457 cm^−1^ were clearly detected in the subtracted spectrum, while a positive peak appeared at higher wavenumbers (3530 cm^−1^). These findings indicate that at the end of the thermochemical transition around 180 °C, the number of free OH groups increased and the number of bounded OH groups decreased.

## 3. Discussion

The above findings are in agreement with the absence of mass loss in the temperature range 180–250 °C. As discussed in the previous section, the increase of C=C bonds (in general double bonds are more stable than single bonds) and the similarity of silybin’s structure with that of char (suggesting easy production of char) can provide an explanation for the increased thermal stability after heating to 180 °C. The experimentally confirmed decrease in the content of highly shifted OH groups (translated to a decreased number of weak chemical bonds) after heating to 180 °C, and the corresponding increase of free OH (translated to an increased number of strong chemical bonds), can provide an additional explanation for the increased thermal stability and the absence of mass loss after 180 °C. Briefly, after heating to 180 °C, some weak chemical bonds in the residue were removed while the number of stronger bonds increased. Thus, the residue was more stable compared to raw silybin and further increase of temperature was required to break its bonds and bring about its further decomposition.

As discussed in the introduction section, clear evidence can be found in the literature that peculiarities in the thermal behaviors of silybin and certain polymers (i.e., the inability to melt without decomposing), are similarly exhibited by other low molecular weight substances with increased capacity for hydrogen bonding. In such systems, the hydrogen bonding has a double effect: (a) it inhibits melting by keeping the molecules close to each other, and (b) it facilitates decomposition by weakening chemical bonds. In addition, it is clear this double effect is self-enhanced, i.e., the stronger the physical interaction, the weaker the chemical bond becomes, and thus the greater is the probability that a substance will exhibit thermochemical transition rather than melting.

## 4. Materials and Methods

Silybin (purity > 98%) was purchased from Santa Cruz Biotechnology. Potassium bromide (KBr) of purity higher than 99.5 %wt. was purchased from Chem-Lab. For the experiments, a Sartorius scale (model B 120S, ±0.0001 g), a Shimadzu differential scanning calorimeter (model DSC-50), a Shimadzu thermogravimetric analyzer (model TGA-50), a Biorad FTS-175 Fourier transform infrared spectrometer (FTIR), a Brucker (model D8 Advance) diffractometer equipped with a Siemens X-Ray tube (Cu, 1.54 Å), and a digital microscope were used.

Silybin was investigated by differential scanning calorimetry (DSC) under nitrogen atmosphere (flow 20 mL/min) from 40 °C to 200 °C with a heating rate of 10 K/min. Two thermogravimetry (TGA) measurements of silybin were performed under nitrogen atmosphere (flow 20 mL/min) with a heating rate of 10 K/min. The first TGA measurement was performed from 40 °C to 600 °C. The second TGA measurement was performed from 40 °C to 180 °C (the curve is not presented). After heating to 180 °C, the silybin sample was removed from the TGA pan and mixed with KBr at a silybin-to-KBr mass proportion of ~1:200, then processed into pellets using a hydraulic press at a pressure of 100 Bar. The raw silybin sample was also processed into KBr pellets, at the same proportion and pressure as the heated sample. FTIR measurements were performed with a resolution of 2 cm^−1^ (64 scans, absorption mode). Baseline alignment and normalization in a scale from 0 to 1 were applied to the obtained spectra. X-ray diffraction (XRD) measurements were taken for the raw silybin as well as silybin heated in the TGA up to 180 °C.

## 5. Conclusions

Differential scanning calorimetry, thermogravimetry, derivative thermogravimetry, infrared spectroscopy, and microscopy were used to study the thermal behavior of silybin. It was shown that the softening of silybin, previously erroneously attributed to neat melting, in fact represents a thermochemical transition. The heat involved in this thermal effect can be expressed in manner other than heat of fusion, by taking into account the decomposed mass and not the overall mass of the sample. This value (specific heat of thermochemical transition) is comparable to the order of magnitude of the dissociation energy of chemical bonds. Mass loss of 4 wt%. occurred during softening. This decomposition caused changes in the chemical structure of silybin that were readily detectable by the darker color of the heated sample and confirmed by the use of infrared spectroscopy. The structure produced during thermochemical transition up to 180 °C was more thermally stable than silybin and provided thermal stability and absence of mass loss up to 250 °C, the temperature where decomposition was initiated. Data available in the literature along with experimental data obtained in this study indicate that the effect of simultaneous softening and decomposition, recently reported for polymers, is broadly exhibited by various forms of matter including polymers, small molecules, and organic or inorganic natural or synthetic substances. In most cases, the substances exhibiting thermochemical transition are characterized by increased hydrogen bond formation in their structures. Hydrogen bonding inhibits melting and enables decomposition through the weakening of chemical bonds.

## Figures and Tables

**Table 1:** Specific heat of the thermal effect at 163.7 °C, estimated by two approaches.

Temperature Range, for Peak 1, °C	Specific Heat of “Fusion”, J/g	Specific Heat of Thermochemical Transition J/g
120–180	112.3	2850

**Table 2:** Wavenumbers and percentage areas of the fitted peaks, presented in Figure 4.

Fitted Peak	Wavenumber, cm^−1^	% AREA
1	2883	not calculated ^1^
2	2939	not calculated ^1^
3	3118	not calculated ^1^
4	3247	46
5	3421	33
6	3458	18
7	3610	3

^1^ These peaks were not taken into account for the normalization of the % areas since they were assigned to vibrations other than O-H stretching.

## Data Availability

Data are available upon request.

## Sample Availability

Samples of the compounds are available from the authors.
